# Deletion of *FgHOG1* Is Suppressive to the *mgv1* Mutant by Stimulating Gpmk1 Activation and Avoiding Intracellular Turgor Elevation in *Fusarium graminearum*

**DOI:** 10.3389/fmicb.2019.01073

**Published:** 2019-05-22

**Authors:** Jingyi Ren, Chengliang Li, Chengyu Gao, Jin-Rong Xu, Cong Jiang, Guanghui Wang

**Affiliations:** ^1^Purdue-NWAFU Joint Research Center, College of Plant Protection, Northwest A&F University, Yangling, China; ^2^Department of Botany and Plant Pathology, Purdue University, West Lafayette, IN, United States

**Keywords:** cell wall integrity, osmoregulation, *Fusarium graminearum*, Gpmk1 MAP kinase, intracellular turgor

## Abstract

*Fusarium* head blight caused by *Fusarium graminearum* is an important disease of wheat and barley. Previous studies have showed that all three MAP kinase genes, *MGV1, FgHOG1*, and *GPMK1*, are involved in regulating hyphal growth, sexual reproduction, plant infection, and stress responses in this pathogen. To determine the relationship between the Mgv1 and FgHog1 pathways, in this study, we generated and characterized the *mgv1 Fghog1* double mutant. Deletion of *FgHOG1* partially rescued the defects of the *mgv1* mutant in vegetative growth and cell wall integrity but had no effects on its defects in plant infection and DON production. The *mgv1 Fghog1* mutant grew faster and was more tolerant to cell wall stressors than the *mgv1* mutant. Swollen compartments and cell burst were observed frequently in the *mgv1* mutant but rarely in the *mgv1 Fghog1* mutant when treated with fungicide fludioxonil or cell wall stressor Congo red. Conversely, the deletion of *MGV1* also alleviated the hyperosmotic sensitivity of the *Fghog1* mutant in vegetative growth. TGY assays indicated increased phosphorylation of FgHog1 in the *mgv1* mutant, and TEY assays further revealed elevated activation of Gpmk1 in the *mgv1 Fghog1* double mutant, particularly under cell wall stress conditions. Overall, our data showed that deletion of *FgHOG1* partially suppressed the defects of the *mgv1* mutant, possibly by affecting genes related to cell wall integrity and osmoregulation *via* the over-activation of Gpmk1 MAP kinase and avoiding intracellular turgor elevation.

## Introduction

*Fusarium* head blight, a devastating disease of wheat and barley, is caused by the filamentous ascomycete *Fusarium graminearum*. In addition to severe yield losses, infested grains are often contaminated with mycotoxins, including deoxynivalenol (DON) and zearalenone (Jiang et al., 2016; Boenisch et al., 2017). *F. graminearum* also causes stalk and ear rots on maize and infects other small grains (Bai and Shaner, 2004; Goswami and Kistler, 2004). It overwinters and produces perithecia on plant debris. Ascospores discharged from perithecia are the primary inoculum to infect flowering wheat heads (Trail, 2009). *F. graminearum* also produces asexual spores known as conidia that are infectious and important for pathogen spreading.

Like other filamentous ascomycetes, *F. graminearum* has three well-conserved mitogen-activated protein (MAP) kinase cascades that are involved in regulating various developmental and infection processes in fungal pathogens (Wang et al., 2011). *MGV1*, the first MAP kinase (MAPK) gene characterized in *F. graminearum*, is orthologous to the cell wall integrity (CWI) MAPK *SLT2* in the budding yeast and *MPS1* in *Magnaporthe oryzae* (Xu et al., 1998; Hou et al., 2002). Deletion of *MGV1* causes severe defects in vegetative growth and plant infection. The *mgv1* mutant is almost non-pathogenic and is significantly reduced in hyphal growth and DON production. *MGV1* is important for CWI, and deletion of *MGV1* results in hypersensitivity to cell wall stress or lytic enzymes. The *mgv1* mutant is blocked in hyphal fusion, and it is female sterile during sexual reproduction (Hou et al., 2002).

The other two MAPK genes, *GPMK1* (*FMK1*) and *FgHOG1*, also have been functionally characterized in *F. graminearum* (Jenczmionka et al., 2003; Zheng et al., 2012). *GPMK1*, an ortholog of yeast *FUS3*/*KSS1* and *M. oryzae PMK1* (Xu and Hamer, 1996), is essential for regulating sexual and asexual production, plant infection processes, and the expression of genes encoding secreted lytic enzymes (Jenczmionka et al., 2003; Urban et al., 2003). The *gpmk1* deletion mutant is reduced in growth rate but blocked in perithecium formation. Whereas Mgv1 and Gpmk1 are two MAPKs with the TEY dual-phosphorylation site, FgHog1, the third MAPK in *F. graminearum* has the TGY motif. FgHog1 is orthologous to yeast Hog1 of the high-osmolarity glycerol (HOG) pathway and *M. oryzae* Osm1 (Dixon et al., 1999; Saito and Tatebayashi, 2004). Deletion of *FgHOG1* results in a minor reduction in vegetative growth but significantly reduced DON production. The *Fghog1* mutant is defective in response to hyperosmotic and oxidative stresses but resistant to fludioxonil fungicides that cause the overstimulation of the HOG pathway and increase in intracellular turgor in fungi (Zheng et al., 2012; Segorbe et al., 2017).

Interestingly, all three MAPK pathways appear to be involved in regulating growth, plant infection, responses to different stresses, and sexual reproduction (Wang et al., 2011). However, their functional relationship and possible cross-talking among different pathways have not been well studied in *F. graminearum*. It was previously reported that the deletion of *MKK1* (the MAPKK of the CWI pathway) significantly reduced the phosphorylation level of FgHog1 (Yun et al., 2014), suggesting a cross talk between the HOG pathway and the CWI pathway. In this study, we generated and characterized the *mgv1 Fghog1* double mutant. Deletion of *FgHOG1* partially rescued the defects of the *mgv1* mutant in vegetative growth, CWI, and responses to elevated temperature but had no effect on its defects in plant infection and DON production. Interestingly, the *mgv1* mutant was hypersensitive to fludioxonil, which also could be partially suppressed by deletion of *FgHOG1*. Furthermore, the *mgv1 Fghog1* mutant had an increased phosphorylation level of Gpmk1. Overall, our results showed that deletion of *FgHOG1* partially suppressed some of the defects of the *mgv1* mutant, possibly by increasing the activation of Gpmk1 and avoiding the upsurge of intracellular turgor.

## Results

### Deletion of *FgHOG1* Partially Suppressed the Growth but Not the Virulence Defect of the *mgv1* Mutant

**FIGURE 1:**
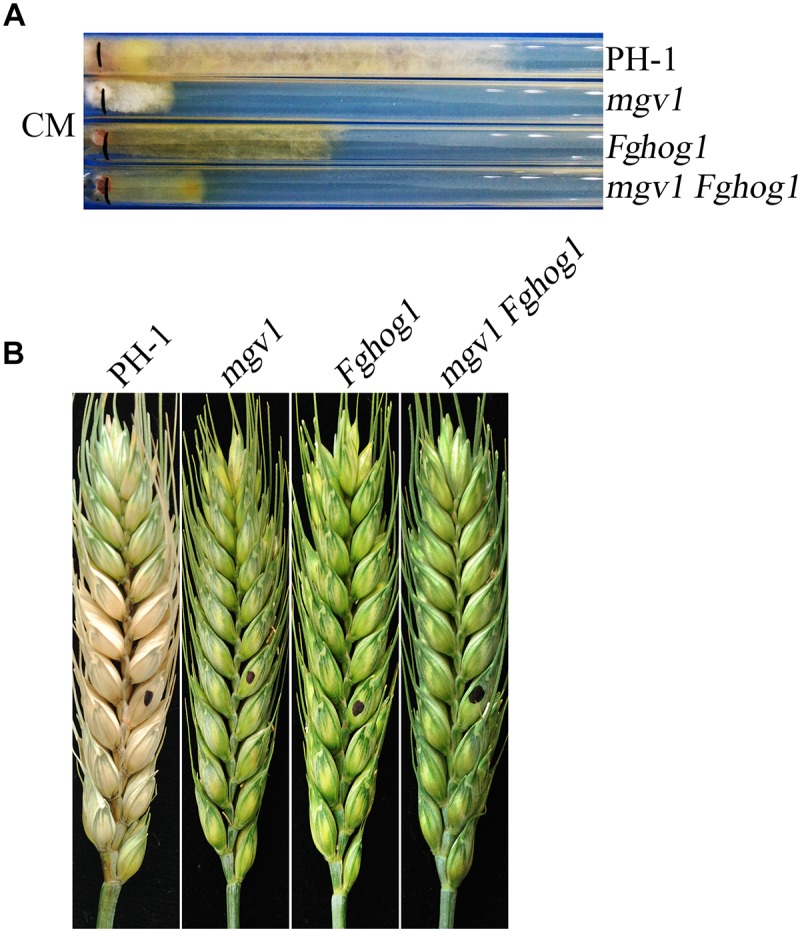
Defects of the *mgv1 Fghog1* mutant in vegetative growth and plant infection. **(A)** Complete medium (CM) cultures of the wild type (PH-1), *mgv1*, *Fghog1*, and *mgv1 Fghog1* mutant strains grown in race tubes at 25°C for 12 days. The *mgv1 Fghog1* double mutant has a faster growth rate than the *mgv1* mutant. **(B)** Wheat heads inoculated with the same set of strains were examined at 14 dpi. Black dots mark the inoculation sites.

To generate the *mgv1 Fghog1* double mutant, the *FgHOG1* gene replacement construct carrying the geneticin resistance marker (*NEO*) was generated and transformed into the *mgv1* mutant. Transformants resistant to both hygromycin B and geneticin were isolated and screened by PCR for the deletion of *FgHOG1* (Supplementary Figure S1). Two *mgv1 Fghog1* mutant strains, MH17 and MH18, were identified, and they had the same phenotypes described below, although only data with strain MH18 were presented. To our surprise, the *mgv1 Fghog1* mutant grew faster than the *mgv1* mutant (Figure 1A). When assayed with race tube cultures grown on CM (complete medium), the *mgv1* mutant grew at approximately 2.7 mm/day, but the growth rate of the *mgv1 Fghog1* mutant was over 4.0 mm/day (Table 1), which was approximately a 48% increase in comparison with *mgv1*. These results indicate that deletion of *FgHOG1* partially suppressed the growth defect of the *mgv1* mutant. However, in infection assays with wheat heads, the *mgv1 Fghog1* mutant, like the *mgv1* and *Fghog1* mutants, rarely caused limited necrosis on glumes but never caused typical symptoms on the inoculated kernels or spread infection to neighboring spikelets (Figure 1B and Table 1). Under the same conditions, the wild type strain PH-1 caused severe head blight symptoms in inoculated wheat heads and had a disease index of over 10 (Figure 1B and Table 1). We also performed DON production assays in TBI cultures and found that the DON was not detectable in the *mgv1 Fghog1* double mutant as well as in the *mgv1* and *Fghog1* single mutants (Table 1). Therefore, deletion of *FgHOG1* in the *mgv1* mutant had no suppressive effect on its virulence defect.

**Table 1:** Phenotype characterization of the mutants used in this study.

Strains	Growth Rate ^a^ (mm/day)	Disease index ^b^	DON production ^c^ (ppm)	Release of protoplasts ^d^ 10^5^ protoplasts/mL)
PH-1 (WT)	8.4 ± 0.6^A∗^	10.3 ± 2.6^A^	124.3 ± 21.5	9.3 ± 2.4^A^
C6 (*mgv1*)	2.7 ± 0.0^C^	0^B^	nd	61.8 ± 3.3^B^
HG15 (*Fghog1*)	8.2 ± 0.4^A^	0^B^	nd	2.7 ± 3.8^C^
MH18 (*mgv1 Fghog1*)	4.0 ± 0.2^B^	0^B^	nd	2.9 ± 8.8^C^


*^a^Growth rate was measured with CM race tube cultures after incubation for 12 days. Average growth rate and standard error (mean ± SE) were calculated from at least three independent measurements. ^*b*^Disease index was rated by the number of symptomatic spikelets at 14 dpi. At least 10 wheat heads were examined in each repeat. ^*c*^DON production was assayed in TBI cultures. nd, not detected. ^*d*^The release of protoplasts was examined after digestion with a mixture of lytic enzymes for 30 min. ^∗^Data from three replicates were analyzed with the protected Fisher least significant difference (LSD) test. Different letters indicate statistically significant difference (*P* = 0.05).*

### The *mgv1 Fghog1* Mutant Is More Tolerant to Cell Wall Stresses Than the *mgv1* Mutant

**FIGURE 2:**
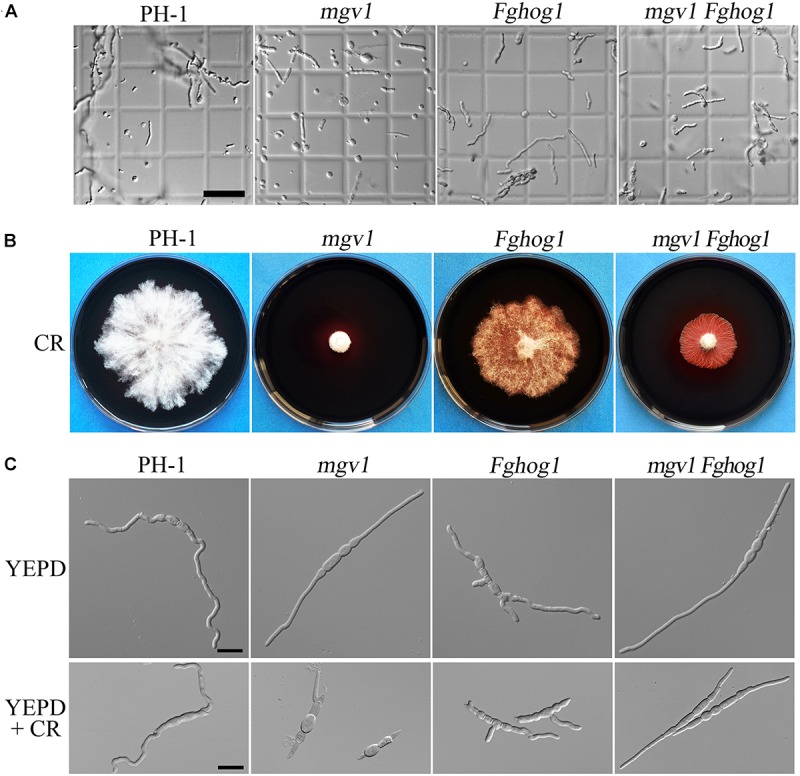
Sensitivities of the *mgv1 Fghog1* mutant to cell wall lytic enzymes and Congo red (CR). **(A)** Germlings harvested from 12 h yeast extract–peptone–dextrose medium (YEPD) cultures of the marked strains were examined after digestion with a mixture of lytic enzymes for 30 min. In comparison with the *mgv1* mutant, the *mgv1 Fghog1* double mutant released fewer protoplasts. Bar = 50 μm. **(B)** Five-day-old cultures of the marked strains grown on CM with 300 μg/ml of CR. The *mgv1 Fghog1* mutant grew faster than the *mgv1* mutant. **(C)** Conidia were examined for germination and germ tube growth after incubation in liquid YEPD with or without 15 μg/ml of CR for 6 h. Bar = 20 μm.

It has been reported that the *mgv1* mutant is hypersensitive to cell wall–degrading enzymes (Hou et al., 2002). To determine whether the inactivation of FgHog1 could recover other defects of the *mgv1* mutant, germlings harvested from 12 h YEPD (yeast extract–peptone–dextrose medium) cultures were treated with a mixture of cell wall lytic enzymes for 30 min. In the *mgv1* mutant, abundant protoplasts were produced, while germlings from the wild type strain PH-1 released few protoplasts (Figure 2A and Table 1), which is consistent with the previous study (Hou et al., 2002). However, under the same conditions, the *Fghog1* and *mgv1 Fghog1* mutants released fewer protoplasts than *mgv1* (Figure 2A and Table 1). These results show that deletion of *FgHOG1* also alleviated the hypersensitivity of the *mgv1* mutant to cell wall lytic enzymes.

To confirm the partial recovery of *mgv1 Fghog1* in cell wall defects, we then assayed hyphal growth on CM with 300 μg/ml Congo red (CR), a compound interfering with the fungal cell wall (Merzendorfer, 2013). The inhibition rate on colony growth by CR was 41.9% in the *mgv1 Fghog1* double mutant, which was significantly lower than that (74.0%) of the *mgv1* mutant (Table 2 and Figure 2B). We also assayed the effects of CR on conidial germination. After incubation in regular YEPD for 6 h, conidia of the double mutant had no obvious defects in germination in comparison with PH-1 or the *mgv1* or *Fghog1* mutant (Figure 2C). However, in the presence of as low as 15 μg/ml CR, the conidial germination rate was only 14% in the *mgv1* mutant, although abnormal swelling was observed in some conidium compartments (Figure 2C and Table 2). Under the same conditions, the rate of conidial germination was not affected in the strains of PH-1 and *Fghog1* mutant, but reduced to 78.8% in the double mutant (Table 2). Germ tubes of the *Fghog1* and *mgv1 Fghog1* mutants also tended to be shorter than those of PH-1 (Figure 2C). These data further indicate that deletion of *FgHOG1* suppressed the CWI defect of the *mgv1* mutant. Abnormal swelling in the *mgv1* mutant may be caused by the stimulation of FgHog1 by CR treatment, leading to an increase in intracellular turgor.

**Table 2:** Different responses of the mutants under various stresses.

Strains	Growth inhibition rate (%)^a^ 300 μg/mL CR	Germination rate (%) ^b^			
		CR (15 μg/mL)	CFW (7.5 μg/mL)	Fludioxonil (5 μg/mL)	NaCl (0.7 M)
PH-1 (WT)	23.9 ± 3.7^C∗^	99.7 ± 0.5^A^	99.2 ± 0.7^A^	38.8 ± 1.8^B^	98.9 ± 0.9^A^
C6 (*mgv1*)	74.0 ± 1.4^A^	14.2 ± 4.8^C^	0.9 ± 0.7^C^	0^C^	97.0 ± 0.2^A^
HG15 (*Fghog1*)	43.7 ± 1.8^B^	99.1 ± 1.5^A^	99.0 ± 1.7^A^	99.2 ± 0.8^A^	94.6 ± 1.8^A^
MH18 (*mgv1 Fghog1*)	41.9 ± 2.9^B^	78.8 ± 3.7^B^	40.4 ± 4.0^B^	98.5 ± 0.1^A^	81.6 ± 0.3^B^


*^a^The growth inhibition rate by Congo red (CR) was estimated by the percentage of the colony diameter of each strain on CM with 300 μg/mL CR in comparison with that of regular CM. ^*b*^Germination rate was measured in YEPD liquid medium with different stresses after incubation for 6 h. Means and standard errors (mean ± SE) were calculated from at least three independent experiments. ^∗^Data from three replicates were analyzed with the protected Fisher least significant difference (LSD) test. Different letters indicate statistically significant difference (*P* = 0.05).*

### Cell Wall Deposition Is Affected in the *mgv1 Fghog1* Mutant

**FIGURE 3:**
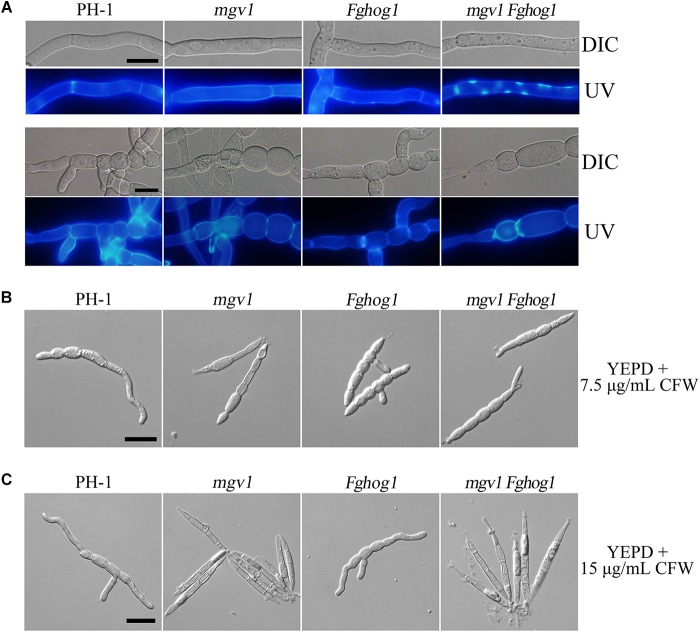
Cell wall deposition is not normal in the *mgv1 Fghog1* mutant. **(A)** Germlings of the wild type, *mgv1*, *Fghog1*, and *mgv1 Fghog1* strains from 12 h YEPD cultures were stained with 50 μg/ml Calcofluor white (CFW) and examined by different interference contrast (DIC) and epifluorescence microscopy. Bright-staining spots were observed in the germ tubes (upper panel) but not in conidium compartments (lower panel) of the *Fghog1* and double mutants. Bar = 10 μm. **(B)** Conidia of the same set of strains were incubated in liquid YEPD with 7.5 μg/ml of CFW for 6 h and were examined by DIC and epifluorescence microscopy. Extreme swelling and empty conidia compartments were observed in the *mgv1* mutant. Bar = 20 μm. **(C)** Conidia of the same set of strains were incubated in liquid YEPD with 7.5 μg/ml of CFW for 6 h and were examined by DIC and epifluorescence microscopy. Most conidia of both *mgv1* and *mgv1 Fghog1* mutants showed empty conidia compartments. Bar = 20 μm.

Calcofluor white (CFW) is a commonly used stain to visualize the fungal cell wall. When germlings harvested from 12 h YEPD cultures were stained with 50 μg/ml CFW, fluorescence signals were evenly distributed along the cell wall and septa in the wild type and *mgv1* mutant (Figure 3A). However, we noticed that CFW staining often resulted in the burst of germ tubes at the tip of the *mgv1* mutant during microscopic examination, suggesting a weakened cell wall at the tips. In the *mgv1 Fghog1* mutant, bright spots of CFW staining were observed in many germlings but not in conidium compartments (Figure 3A). Weaker and fewer CFW staining spots also were observed in germlings of the *Fghog1* mutant (Figure 3A). These data indicate that deletion of *FgHOG1* caused uneven deposition of cell wall, especially in the *mgv1* mutant.

We also assayed the effect of CFW on conidial germination. In the presence of 7.5 μg/ml CFW, the conidial germination rate of the *mgv1* mutant was lower than 1%, while that of the *mgv1 Fghog1* double mutant was increased to 40% (Table 2). Interestingly, the *mgv1* mutant had empty conidium compartments besides swollen compartments (Figure 3B). Under the same conditions, empty compartments in conidia were rarely observed in the *mgv1 Fghog1* double mutant (Figure 3B). Empty compartments observed in the *mgv1* mutant may be caused by cell burst resulting from a weakened cell wall and elevated intracellular turgor. However, when we used a higher concentration (15 μg/ml) of CFW to treat the conidia, we found that all conidia of both the *mgv1* and *mgv1 Fghog1* mutants were unable to germinate and had empty conidium compartments (Figure 3C). These data indicate that the *mgv1 Fghog1* double mutant was still defective in CWI, although deletion of *FgHOG1* partially rescued the cell wall defect of the *mgv1* mutant.

### The Sensitivity of the *mgv1* Mutant to Elevated Temperatures Also Is Suppressed by Deletion of *FgHOG1*

**FIGURE 4:**
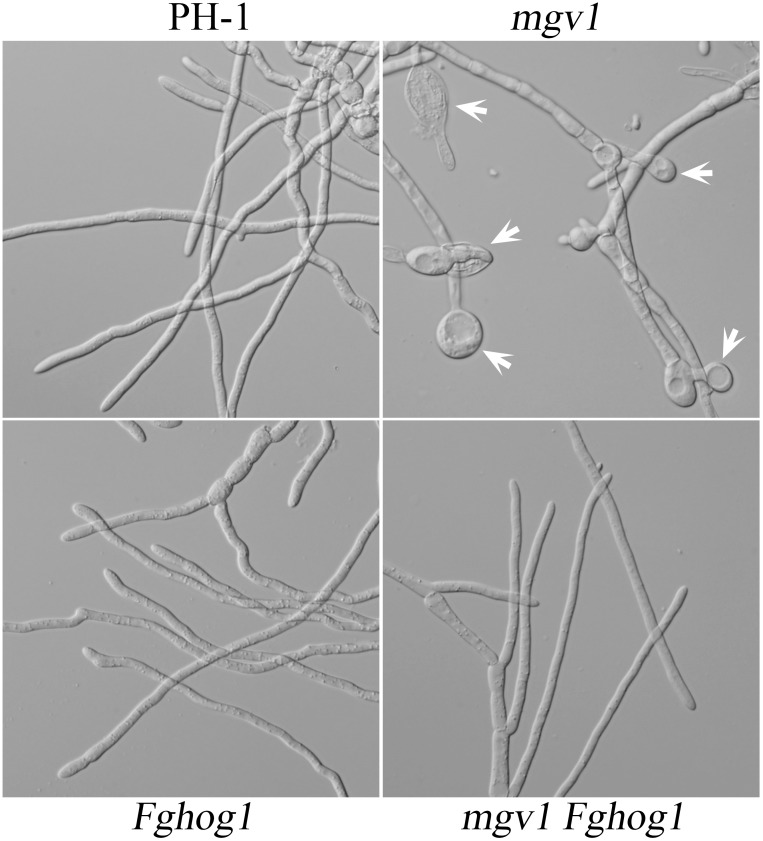
Sensitivity of *mgv1* and *mgv1 Fghog1* mutants to elevated temperature. Germlings of the marked strains from 18 h YEPD cultures were shifted to 32°C and incubated for another 6 h. Swollen hyphal tips (marked with arrows) were abundant in the *mgv1* mutant but not in other strains. However, the germ tubes of *Fghog1* tended to curve and had irregular swelling that was different from the swollen hypha tips of *mgv1*. Bar = 20 μm.

Another phenotype of the *mgv1* mutant related to its CWI defect is increased sensitivity to elevated temperatures (Hou et al., 2002). When germlings from 18 h YEPD cultures grown at 25°C were shifted to 32°C and incubated for another 6 h, swollen hyphal tips and empty hyphal compartments were often observed in the *mgv1* mutant (Figure 4). However, we failed to observe swollen hyphal tips and empty hyphal compartments in germlings of the *mgv1 Fghog1* mutant (Figure 4), suggesting that deletion of *FgHOG1* also could partially suppress the sensitivity of *mgv1* to elevated temperatures.

### The *mgv1 Fghog1* Mutant Is More Tolerant to Fludioxonil Than the *mgv1* Mutant

**FIGURE 5:**
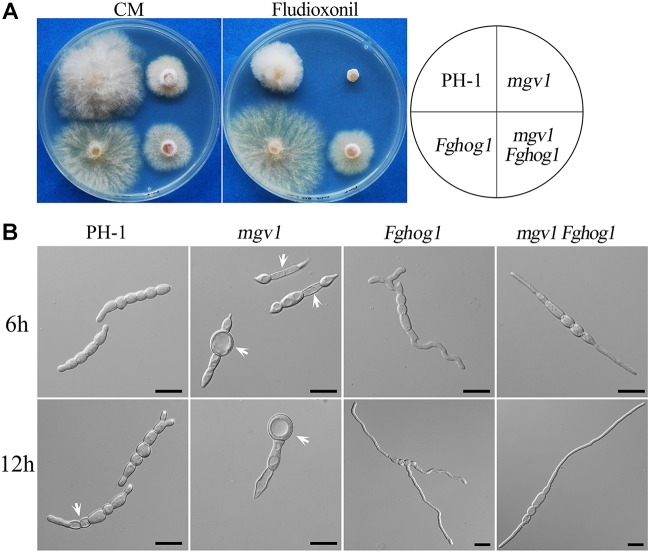
Suppression of the hypersensitivity of *mgv1* to fludioxonil by deletion of *FgHOG1*. **(A)** Three-day-old cultures of PH-1, *mgv1*, *Fghog1*, and *mgv1 Fghog1* strains grown on CM with 0.1 μg/ml fludioxonil at 25°C. The double mutant was able to grow but formed smaller colonies than the *Fghog1* mutant. **(B)** Conidia of the same set of strains were incubated in YEPD with 5 μg/ml fludioxonil for 6 and 12 h before examination. Bar = 20 μm. Treatment with such a low concentration of fludioxonil caused the swelling and burst of conidium compartments in the *mgv1* mutant but not in other strains.

Like in other filamentous fungi, the FgHog1 pathway is involved in resistance to fludioxonil fungicides (Segorbe et al., 2017). In the presence of 0.1 μg/ml fludioxonil, colony growth was significantly inhibited in the wild type and totally blocked in the *mgv1* mutant, but not affected in the *Fghog1* mutant (Figure 5A). Under the same conditions, the *mgv1 Fghog1* double mutant could grow and form colonies that were smaller than those of the *Fghog1* mutant (Figure 5A). These results indicate that deletion of *FgHOG1* could confer tolerance to fludioxonil in the *mgv1* mutant. Interestingly, unlike the *Fghog1* mutant, the double mutant grew faster on regular CM than on CM with fludioxonil, suggesting that the *mgv1 Fghog1* mutant is still sensitive to fludioxonil. Therefore, Mgv1 also plays a role in tolerance against fludioxonil in *F. graminearum*, possibly by strengthening the cell wall.

We then assayed the effects of fludioxonil treatment on conidial germination and germ tube growth. After incubation for 6 h in YEPD with 5 μg/ml fludioxonil, conidia swelled but germinated with a low rate (38.8%) in the wild type (Table 2). In the *mgv1* mutant, no germination was observed, but some of its conidium compartments had ballooned (large swelling) or became empty (Figure 5B and Table 2). The presence of fludioxonil had no obvious effects on germination in the *Fghog1* and *mgv1 Fghog1* mutants (Figure 5B and Table 2). After incubation for 12 h, majority of conidia produced short germ tubes, and some conidium compartments became empty in the wild type. In the *mgv1* mutant, more conidium compartments had ballooned or became empty, but most conidia failed to germinate (Figure 5B). These results indicate that the *mgv1* mutant had increased sensitivity to fludioxonil compared to the wild type, and the increase was suppressed by deletion of *FgHOG1*. Because fludioxonil is fungicidal by overstimulating the HOG pathway and resulting in a rise in intracellular turgor (Zhang et al., 2002), the increased sensitivity of the *mgv1* mutant may be directly related to its cell wall defects.

**FIGURE 6:**
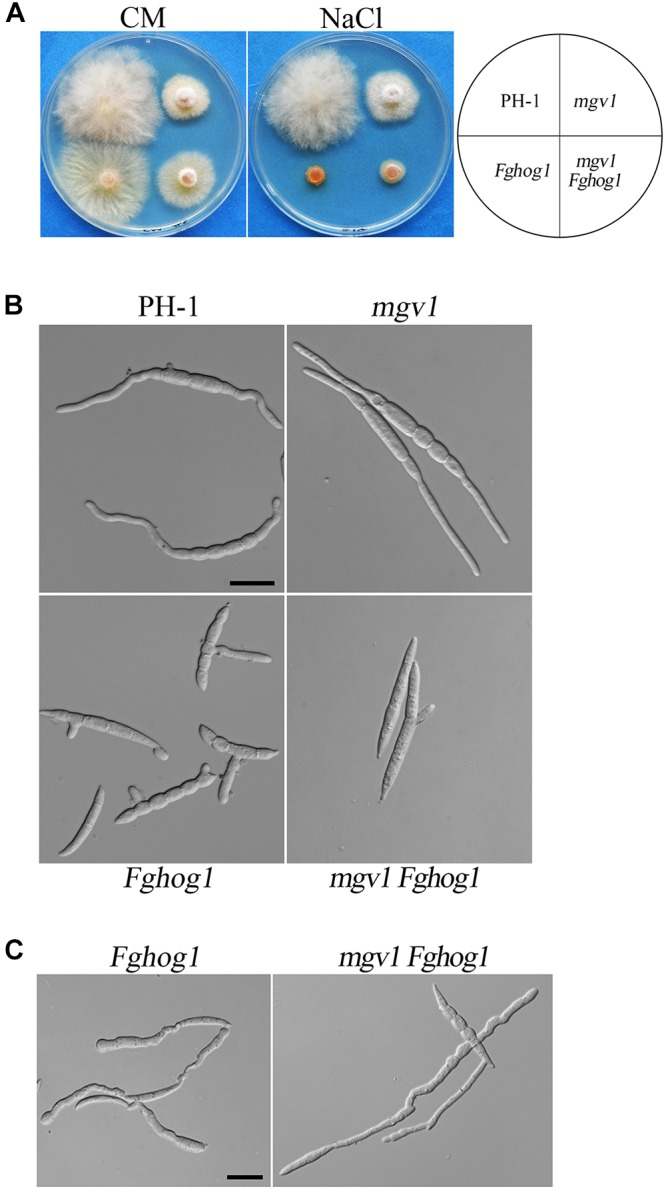
Deletion of *MGV1* caused different effects on vegetative growth and conidial germination of the *Fghog1* mutant under hyperosmotic stress. **(A)** Three-day-old cultures of PH-1, *mgv1*, *Fghog1*, and *mgv1 Fghog1* strains grown on CM plates with or without 0.2 M NaCl. **(B)** Conidia of the same sets of strains were examined after incubation for 6 h in YEPD with 0.7 M NaCl. Bar = 20 μm. **(C)** Conidia of the Fghog1 mutant and mgv1 Fghog1 mutants incubated for 12 h in YEPD with 0.7 M NaCl were examined by DIC. Bar = 20 μm.

**The *mgv1Fghog1* mutant is more tolerant to hyperosmotic stress than the *Fghog1* mutant in vegetative growth but not in conidial germination:** Because FgHog1 MAPK is involved in regulating the response to hyperosmotic stress, we then assayed the effects of 0.2 M NaCl on the growth of the *mgv1* and *mgv1 Fghog1* mutants. As shown in Figure 6A, both the *Fghog1* and *mgv1 Fghog1* mutants were significantly inhibited in vegetative growth on a CM plate with 0.2 M NaCl. Interestingly, the *mgv1 Fghog1* double mutant formed a bigger colony than the *Fghog1* mutant, which was contrary to the situation on a regular CM plate (Figure 6A), indicating that the deletion of *MGV1* partially alleviated the hypersensitivity of the *Fghog1* mutant against hyperosmotic stress. We also noticed that the *mgv1* mutant grew faster on a CM plate supplemented with 0.2 M NaCl than on a regular CM plate (Supplementary Figure S2). When inoculated in liquid YEPD with 0.7 M NaCl for 6 h, both the *mgv1* and the *Fghog1* mutant germinated as efficiently as the wild type PH-1 (Table 2). In comparison with the wild type, the *Fghog1* mutant but not the *mgv1* mutant had shorter germ tubes and tended to have slightly swollen tips (Figure 6B). Intriguingly, under same conditions, the double mutant had a lower conidial germination rate (81.6%) than *mgv1* (97.0%) and *Fghog1* (94.6%) (Figure 6B and Table 2). However, after incubation for 12 h, majority of *mgv1 Fghog1* germ tubes were longer than those of the *Fghog1* mutant (Figure 6C). These data indicate that the deletion of *MGV1* reduced the conidial germination rate of the *Fghog1* mutant under hyperosmotic stress but alleviated its hyperosmotic sensitivity in vegetative growth.

### Deletion of *FgHOG1* Increases the Phosphorylation of Gpmk1 in the *mgv1* Mutant

**FIGURE 7:**
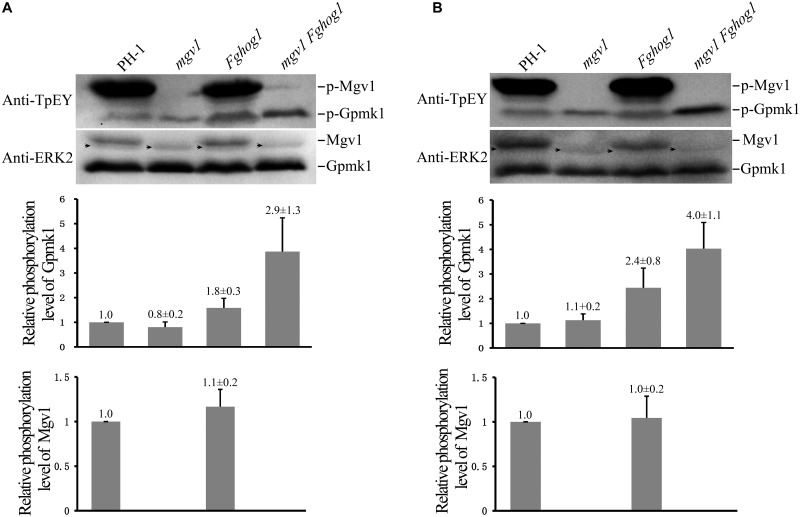
Deletion of *FgHOG1* increases the phosphorylation of Gpmk1 in the *mgv1* mutant. **(A)** Western blots of total proteins isolated from vegetative hyphae of the marked strains harvested from 18 h regular YEPD cultures were detected with the anti-TpEY phosphorylation-specific antibody or anti-ERK2 MAPK antibody (upper panel). As expected, the Western bands of Mgv1 and phosphorylated Mgv1 (p-Mgv1) were not detected from the *mgv1* and *mgv1 Fghog1* mutants. The relative phosphorylation level of Gpmk1 (*y*-axis) was estimated as the ratio of the phosphorylated Gpmk1 band detected with an anti-TpEY antibody to the total Gpmk1 band detected with the anti-ERK2 antibody for individual mutant strains, and compared to that of the wild type (middle panel). The relative phosphorylation level of Mgv1 (*y*-axis) was estimated as the ratio of the phosphorylated Mgv1 band detected with an anti-TpEY antibody to the total Mgv1 band detected with the anti-ERK2 antibody, and compared to that of the wild type (lower panel). Black arrows denote non-specific bands. Error bars represent standard deviations from three independent experiments. **(B)** 18 h vegetative hyphae of the same set of strains were treated with 10 μg/ml CR for 10 min and used for Western blot analyses with anti-TpEY and anti-ERK2 antibodies. Black arrows denote non-specific bands. Error bars represent standard deviations from three independent experiments.

The phosphorylation of Gpmk1 and Mgv1 at the TEY motif could be detected with a commercially available anti-TpEY phosphorylation-specific antibody (Zhang et al., 2017). On Western blots of total proteins isolated from vegetative hyphae harvested from 18 h regular YEPD cultures, the Gpmk1 and phosphorylated Gpmk1 bands were detected in all four strains, while the Mgv1 and phosphorylated Mgv1 bands were only detectable in the wild type and the *Fghog1* mutant (Figure 7A). In comparison with the wild type, the phosphorylation of Gpmk1 was increased about 1.8- and 2.9-fold in the *Fghog1* and *mgv1 Fghog1* mutants, respectively (Figure 7A). The phosphorylation level of Gpmk1 also was higher in the double mutant than in the *Fghog1* mutant. We also assayed the effects of CR treatment on MAPK phosphorylation. Hyphae harvested from 18 h YEPD cultures were further treated with 10 μg/ml CR for 10 min. On Western blots detected with the anti-TpEY antibody, phosphorylation of Gpmk1 was increased approximately 2.4- and 4.0-fold in *Fghog1* and *mgv1 Fghog1* mutants, respectively, in comparison with the wild type (Figure 7B). However, under normal and CR stress conditions, the wild type and the *Fghog1* mutant displayed similar phosphorylation levels of Mgv1 (Figure 7A,B). These results indicate that deletion of both *FgHOG1* and *MGV1* resulted in an increase in the activation of the Gpmk1 MAPK under normal or cell wall stress conditions, which may contribute to the suppressive effects of *FgHOG1* deletion on the *mgv1* mutant.

### Deletion of *MGV1* Increased the Phosphorylation of FgHog1 in Response to Fludioxonil

**FIGURE 8:**
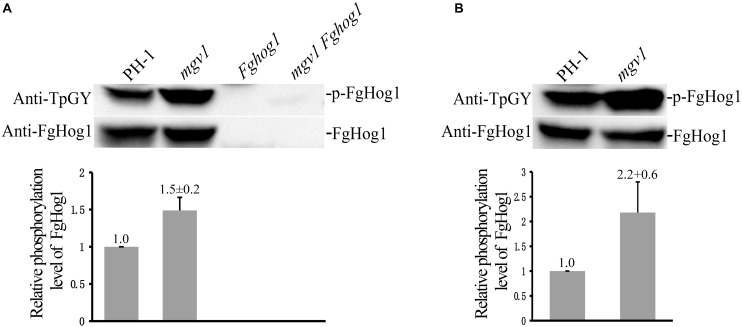
Assays for the phosphorylation of FgHog1. **(A)** Western blots of total proteins isolated from vegetative hyphae harvested from 18 h regular YEPD cultures were detected with the anti-TpGY phosphorylation-specific antibody or anti-FgHog1 antibody. The FgHog1 band was not detectable in the *Fghog1* and *mgv1 Fghog1* mutants. In the *mgv1* mutant, the phosphorylation of FgHog1 was increased by approximately 50% in comparison with the wild type. **(B)** Western blots of total proteins isolated from vegetative hyphae of marked strains treated with 2 μg/ml fludioxonil (Flu) for 10 min. The phosphorylation of FgHog1 in the *mgv1* mutant was two times higher than that in the wild type.

We also assayed the phosphorylation of FgHog1 with the anti-TpGY–specific antibody because FgHog1 is the only MAPK with the TGY dual-phosphorylation site in *F. graminearum*. On Western blots with total proteins isolated from 18 h regular YEPD cultures, as expected, the phosphorylated FgHog1 band was not detected with the anti-TpGY antibody in the *Fghog1* and *mgv1 Fghog1* mutants (Figure 8A). The phosphorylation level of FgHog1 was increased about 1.5-fold in the *mgv1* mutant (Figure 8A). We also conducted Western blot analysis with proteins isolated from 18 h hyphae that were treated with 2 μg/ml fludioxonil for 10 min. In comparison with the wild type, fludioxonil treatment increased the phosphorylation of FgHog1 over 2-fold in the *mgv1* mutant (Figure 8B). These results indicate that deletion of *MGV1* had a positive effect on FgHog1 phosphorylation under both normal and fludioxonil conditions. The overstimulation of FgHog1 observed in the *mgv1* mutant may lead to elevated turgor pressure.

## Discussion

In eukaryotic organisms, MAPK signaling pathways play important roles in sensing and responding to various external signals and overcoming host immunity defenses (Tong and Feng, 2019). The budding yeast has five MAPK pathways that regulate mating, invasive growth, CWI, HOG, and ascospore formation. Except for ascosporogenesis-specific MAPK, three MAPK cascades, consisting of MAPKs (Kss1/Hog1/Slt2), MAPK kinases (Ste7/Psb2/Mkk1), and MAPK kinase kinases (Ste11/Ssk2/Bck1), are conserved in pathogenic ascomycetes (Zhao et al., 2007). The regulatory role of each cascade comprises conserved and special aspects. In pathogenic fungi, the Kss1 MAPK is important for plant infection. This pathway is required for appressorium formation in all appressorium-forming pathogens studied, including *M. oryzae*, *Botrytis cinerea*, and *Verticillium dahliae*, and also plays important roles in plant penetration and invasive growth in various non-appressorium-forming pathogens (Jiang et al., 2018). The HOG pathway regulates cellular responses to high osmolarity, oxidation/heat shock, pathogenicity, and phenylpyrrole fungicides (Rispail et al., 2009). The Slt2 orthologs are well conserved in filamentous fungi, but they exhibit distinct biological functions among different species. For instance, the orthologs of Slt2 are well known to regulate the integrity of cell walls in budding yeast, *M. oryzae*, *Ustilago maydis*, and *Aspergillus* species (Xu et al., 1998; Carbo and Perez-Martin, 2010; Rodicio and Heinisch, 2010; Yoshimi et al., 2016) but not in *B. cinerea* and *Colletotrichum lagenarium* (Kojima et al., 2002; Rui and Hahn, 2007). Moreover, Slt2-type MAPKs are required for fungal virulence in *Candida albicans* and *M. oryzae* but not in *Aspergillus fumigatus* (Xu et al., 1998; Monge et al., 2006; Valiante et al., 2009). In nematode-trapping fungi, the Slt2 is also involved in mycelial trap formation (Zhen et al., 2018).

Like many other filamentous ascomycetes, *F. graminearum* has three well-conserved MAPK pathways that have distinct and overlapping functions in growth, development, and pathogenesis (Hou et al., 2002; Jenczmionka et al., 2003; Urban et al., 2003; Zheng et al., 2012). For vegetative growth, the CWI pathway is more important than the other MAPK pathways. Different from the *Fghog1* and *gpmk1* mutants, the *mgv1* deletion mutant has severe growth defects (Hou et al., 2002). In filamentous ascomycetes such as *B. cinerea*, *Neurospora crassa*, and *Alternaria alternata*, disruption of the Slt2 ortholog also caused defects in vegetative growth (Rui and Hahn, 2007; Park et al., 2008; Yago et al., 2011). In this study, we showed that the *mgv1 Fghog1* double mutant grew faster than the *mgv1* mutant, although it was still non-pathogenic. The suppressive effect of *FgHOG1* deletion on *mgv1* growth indicated that these two MAPK pathways may cross talk during vegetative growth in *F. graminearum*. In fact, *FgHOG1* may also play a role in normal hyphal growth because the *Fghog1* mutant itself was slightly reduced in growth rate. The difference in the effects of *FgHOG1* deletion on *mgv1* growth and virulence may be related to the fact that the *Fghog1* mutant itself had severe defects in plant infection, although it was only slightly reduced in vegetative growth.

The *mgv1* mutant is hypersensitive to cell wall stressors and lytic enzymes (Hou et al., 2002). Interestingly, the *mgv1 Fghog1* double mutant was less sensitive than *mgv1* to cell wall lytic enzymes, CR, and CFW. In *Fusarium oxysporum*, the double mutant of these two MAPK genes also showed alleviated sensitivity to cell wall stressor CFW but not to CR (Segorbe et al., 2017). In *F. graminearum*, it is possible that some of the genes important for CWI may be downregulated in the *mgv1* mutant but partially rescued by deletion of *FgHOG1* (directly or indirectly). In yeast, a number of genes important for cell wall synthesis and assembly are affected by deletion of *SLT2* (Levin, 2011). It will be important to assay whether the *mgv1* mutant has similar defects in the regulation of cell wall–related genes and whether deletion of *FgHOG1* impacts their expression in *F. graminearum*.

In comparison with the wild type, the *mgv1* mutant had increased sensitivity to fludioxonil during conidial germination. Fungicide fludioxonil can increase the cytoplasmic solute concentration and intracellular turgor pressure by overstimulating the HOG pathway (Ochiai et al., 2007; Jiang et al., 2011). Deletion of *FgHOG1* also improved the resistance of the *mgv1* mutant to fludioxonil. Consistent with this, in *F. oxysporum*, the *mpk1 hog1* double mutant (equivalent to the *mgv1 Fghog1* mutant in *F. graminearum*) also has higher resistance to fludioxonil than the *mpk1* mutant (Segorbe et al., 2017). In *F. graminearum*, fludioxonil treatment likely increased the intracellular turgor pressure in the *mgv1* mutant, resulting in the swelling and burst of conidia and hyphae due to its cell wall defects. Deletion of *FgHOG1* will avoid the fungicidal effect of fludioxonil on increasing intracellular turgor pressure. We noticed that the *mgv1 Fghog1* double mutant also had increased resistance to elevated temperatures. In the budding yeast, exposure to elevated temperatures results in the accumulation of trehalose in the cytoplasm (Neves and Francois, 1992) and triggers water influx into cell, resulting in an increase in intracellular turgor pressure (Levin, 2005). Therefore, some of the suppressive effects of *FgHOG1* deletion on *mgv1* may be simply related to avoiding an increase in intracellular turgor under certain stress conditions that is regulated by the FgHog1 MAPK pathway in *F. graminearum*.

This hypothesis is also supported by our result that the phosphorylation level of FgHog1 was significantly increased in the *mgv1* deletion mutant, which could at least partially contribute to the defect of the *mgv1* mutant. In line with this, inactivation of Slt2 in *S. cerevisiae* also triggers the activation of Hog1 by downregulating a phosphatase Ptp2 that is involved in dephosphorylation of Hog1 (Chang et al., 2013). The similar mechanism may also exist in *F. graminearum*. Intriguingly, the deletion of *FgMKK1* (the upstream MAPKK of *MGV1*) significantly inhibits the activation of FgHog1 (Yun et al., 2014). Given that the *Fgmkk1* mutant exhibited increased tolerance to fludioxonil and hypersensitivity to hyperosmotic stresses (Yun et al., 2014), which is contrary to the *mgv1* mutant, we think the FgMkk1 and Mgv1 should play distinct roles in the cross talk between the HOG pathway and the CWI pathway.

While deletion of *FgHOG1* was suppressive to most of the defects of the *mgv1* mutant, deletion of *MGV1* also appeared to slightly alleviate the hyperosmotic sensitivity of the *Fghog1* mutant in vegetative growth. It has been reported that in *A. fumigatus*, *U. maydis*, and *Fusarium verticillioides*, the *BCK1*, *MKK1*, and *SLT2* deletion mutants with impaired cell walls had increased tolerance to osmotic stresses (Valiante et al., 2009; Carbo and Perez-Martin, 2010; Zhang et al., 2015). In addition, we had proved that deletion of *FgHOG1* only partially enhanced the cell wall and the *mgv1 Fghog1* was still defective in CWI. Therefore, the alleviated sensitivity of the *mgv1 Fghog1* double mutant to hyperosmotic stress could be attributed to its cell wall defect. To our surprise, the deletion of *MGV1* reduced the conidial germination rate of the *Fghog1* mutant in YEPD with 0.7 M NaCl. We noticed that the majority of conidia of the *mgv1 Fghog1* mutant were unable to swell under this hyperosmotic stress condition, suggesting its failure to form enough intracellular turgor pressure. Accumulation of cytoplasmic solute could cause huge intracellular turgor pressure that is essential for conidial swelling and germination. Undoubtedly, more cytoplasmic solute could be required to generate identical intracellular turgor pressure under hyperosmotic stress conditions. It has been reported that glycerol content increased intensively at the initial swelling stage of germination, and the glycerol is synthesized from storage carbohydrates trehalose and mannitol (Morozova et al., 2002). However, it was reported that the disruption of Slt2 leads to reduced accumulation of mannitol and trehalose in *Beauveria bassiana* (Chen et al., 2014). Thus, we speculated that the reduced conidial germination rate of the *mgv1 Fghog1* double mutant under hyperosmotic stress may be attributable to both the absence of Hog1-mediated glycerol biosynthesis and the impaired storage of trehalose and mannitol in conidia.

When assayed with an anti-TpEY phosphorylation-specific antibody, we found that the *mgv1 hog1* double mutant had an increased Gpmk1 phosphorylation level in normal culture conditions or under stress conditions. Consistent with this, the *mpk1 hog1* double mutant also has an over-activation of Fmk1 (ortholog of Gpmk1) in *F. oxysporum* (Segorbe et al., 2017). Although the CWI pathway has been considered as the main signaling pathway responsible for CWI, other signaling pathways have also been implicated in maintaining cell wall construction (Boorsma et al., 2004; Garcia et al., 2004; Munro et al., 2007). In the budding yeast, the Kss1 MAPK also is involved in the STE vegetative growth (SVG) pathway that promotes vegetative growth *via* regulating the cell wall biosynthesis (Lee and Elion, 1999; Cullen et al., 2000). In *C. albicans*, the Cek1 MAPK signaling pathway also plays an important role in cell wall biogenesis (Eisman et al., 2006). Gpmk1 is an ortholog of Kss1 and Cek1 in *F. graminearum*. It is likely that an increase in the activation of the Gpmk1 MAPK in the *mgv1 Fghog1* mutant will affect the regulation of cell wall generation, which contributes to the suppressive effects of *FgHOG1* deletion on the defects of the *mgv1* mutant. Increased activation of Gpmk1 in the *Fghog1* mutant, especially under hyperosmotic stress conditions, has been observed in an earlier study (Zheng et al., 2012). Although its exact function in CWI and hyphal growth is not clear, the *gpmk1* mutant also displayed a vegetative growth defect (Wang et al., 2011). Therefore, it will be important to characterize the role of Gpmk1 in hyphal growth and cell wall biosynthesis and its relationship with *Mgv1* and *FgHog1* in *F. graminearum*.

## Materials and Methods

### Fungal Strains and Culture Conditions

The wild type strain PH-1 and all the mutants of *F. graminearum* were routinely cultured at 25°C on potato dextrose agar (PDA) (200 g potato, 20 g glucose, and 20 g agar in 1 L water) or CM (10 g glucose, 2 g peptone, 1 g yeast extract, 1 g casamino acids, 6 g NaNO_3_, 0.5 g KCl, 0.5 g MgSO_4_, and 1.5 g KH_2_PO_4_ in 1 L water, pH 6.5) (Wang et al., 2018). Growth rate was measured with race tube cultures (Liu et al., 2015). Conidiation in CMC (15 g carboxymethyl cellulose, 1 g yeast extract, 0.5 g MgSO_4_, 1 g NH_4_NO_3_, and 1 g KH_2_PO_4_ in 1 L water) cultures and conidial germination in YEPD (0.3% yeast extract powder, 1% peptone, and 2% glucose) were assayed as described (Wang et al., 2012; Zheng et al., 2012). For protein extraction, 18 h germlings were harvested from liquid YEPD cultures.

### Generation of the *mgv1 Fghog1* Double Mutant

The *mgv1* and *Fghog1* single mutants were generated in previous studies (Hou et al., 2002; Zheng et al., 2012). To generate the double mutant, the split-marker approach (Catlett et al., 2003) was used to delete the *FgHOG1* gene in the *mgv1* mutant. The 0.91-kb upstream and 0.56-kb downstream flanking fragments of *FgHOG1* gene were amplified with primer pairs HOG1/1F + 2R and HOG1/3F + 4R, respectively. The resultant PCR products were fused with the geneticin resistance cassette amplified with primers GEN/F + GE/R and EN/F + GEN/R from the pFL2 vector (Zhou et al., 2011) and transformed into protoplasts of the *mgv1* mutant as described (Wang et al., 2012). G418 (Sigma-Aldrich, St. Louis, MO, United States) was added to the final concentration of 400 mg/mL for transformant selection (Wang et al., 2018). Transformants resistant to G418 were screened by PCR with primer pairs 5F + 6R, G850 + G852, 7F + G856R, and G855F + 8R to confirm the deletion of *FgHOG1*.

### Assays for Defects in Responses to Different Stresses

The final concentration of 300 μg/ml CR, 0.2 M NaCl, or 0.1 μg/ml fludioxonil was added to the CM to assay for colony growth at 25°C as described (Zhang et al., 2016), and morphology in each plate was examined and photographed after incubation for the time indicated in the figure legends. To assay conidial germination, the final concentration of 15 μg/ml CR, 7.5 μg/ml CFW, 0.7 M NaCl, or 5 μg/ml fludioxonil was added to freshly harvested conidia resuspended in YEPD (10^6^ spores/ml) and incubated for 6 or 12 h at 25°C. In order to check the chitin distribution, germlings harvested from 12 h YEPD cultures were stained with 50 μg/ml CFW for 5 min. Germlings harvested from 18 h YEPD cultures grown at 25°C were shifted to 32°C and incubated for another 6 h to assay the effect of elevated temperature. For CWI assays, germlings harvested from 12 h YEPD cultures were digested with the cell wall lytic solution (0.1 g lysing enzymes, 0.5 g Driselase, and 10 mg Lyticase in 20 ml of 1.2 M KCl) at 30°C for 30 min before examination for protoplasts (Hou et al., 2002; Yun et al., 2014). Observations were conducted under an Olympus BX-51 microscope (Olympus, Tokyo, Japan), and photographs were taken in different interference contrast (DIC) or epifluorescence modes. Each experiment was repeated at least three times independently.

### Plant Infection and DON Production Assays

For plant infection assays, conidia harvested from 5-day-old CMC cultures by filtration through Miracloth were resuspended to a concentration of 1.0 × 10^5^ spores/ml in sterile distilled water. Flowering wheat heads of cultivar Xiaoyan 22 were drop-inoculated with 10 μl of conidia suspensions at the fifth spikelet from the base of the spike as described (Gale et al., 2002). There were at least 10 replicates for each strain. Inoculated wheat heads were covered by a plastic bag for 2 days to keep humidity. Wheat spikelets with typical *Fusarium* head blight symptoms were examined and recorded 14 days post-inoculation (dpi) to estimate the disease index. For each strain, a DON production assay in TBI cultures was performed as described (Gardiner et al., 2009), with a competitive ELISA-based DON plate kit (Beacon Analytical Systems, Saco, ME, United States). This experiment was repeated three times.

### Western Blot Analysis

Total proteins were isolated from hyphae harvested from 18 h YEPD cultures as described (Zhang et al., 2018). For assays with different stresses, hyphae harvested from 18 h YEPD were further incubated with 10 μg/ml CR or 2 μg/ml fludioxonil for another 10 min before protein extraction. Proteins were separated on a 10% SDS-PAGE gel and transferred to nitrocellulose membranes as described (Zhang et al., 2017). The PhophoPlus p44/42 MAPK antibody kit (Cell Signaling Technology, United States) was used to detect the phosphorylation of Gpmk1 and Mgv1 as described (Zhang et al., 2017). Phosphorylation of FgHog1 was assayed with the Phopho p38 MAPK antibody kit (Cell Signaling Technology, United States). The expression level of Gpmk1, Mgv1, and FgHog1 was detected with the anti-Erk2 and anti-FgHog1 polyclonal antibodies. Band densities were analyzed with the Image Lab^TM^ software. Quantitative changes in the phosphorylation levels of Gpmk1, Mgv1, and FgHog1 were analyzed with the Image Lab^TM^ software. Each experiment was repeated four times, independently.

## Author Contributions

GW, CJ, and J-RX conceived and designed the experiments. JR, CL, and CG performed the experiments. GW and CJ analyzed the data. GW wrote the manuscript. J-RX and CJ improved the manuscript.

## Conflict of Interest Statement

The authors declare that the research was conducted in the absence of any commercial or financial relationships that could be construed as a potential conflict of interest.

## Supplementary Material

FIGURE S1 PCR assays for the confirmation of the deletion of *FgHOG1* under the *mgv1* mutant background. **(A)** The *FgHOG1* locus and gene replacement construct. The FgHOG1 and NEO genes are marked with empty and black arrows, respectively. **(B)** A total of 18 G418-resistant *mgv1 Fghog1* transformants (MH1 to MH18) were screened by PCR with primers 5F and 6R. **(C)** MH14, MH17, and MH18 were further verified by PCR with four pairs of primers, L1 (5F + 6R), L2 (G850 + G852), L3 (7F + G856R), and L4 (G855F + 8R).

FIGURE S2 The *mgv1* mutant grew faster on CM containing 0.2 M NaCl than on regular CM. **(A)** Three-day-old cultures of *mgv1* mutant grown on CM with and without 0.2 M NaCl. The *mgv1* mutant grew faster on CM containing 0.2 M NaCl than on regular CM. **(B)** The diagram shows the growth rate of *mgv1* mutant on CM with and 0.2 M NaCl.
