# Supplementary figures and images for: Deletion of FgHOG1 Is Suppressive to the mgv1 Mutant by Stimulating Gpmk1 Activation and Avoiding Intracellular Turgor Elevation in Fusarium graminearum
Source: Front Microbiol. 2019 May 22;10:1073. doi: 10.3389/fmicb.2019.01073 (PMC6538775; doi:10.3389/fmicb.2019.01073)

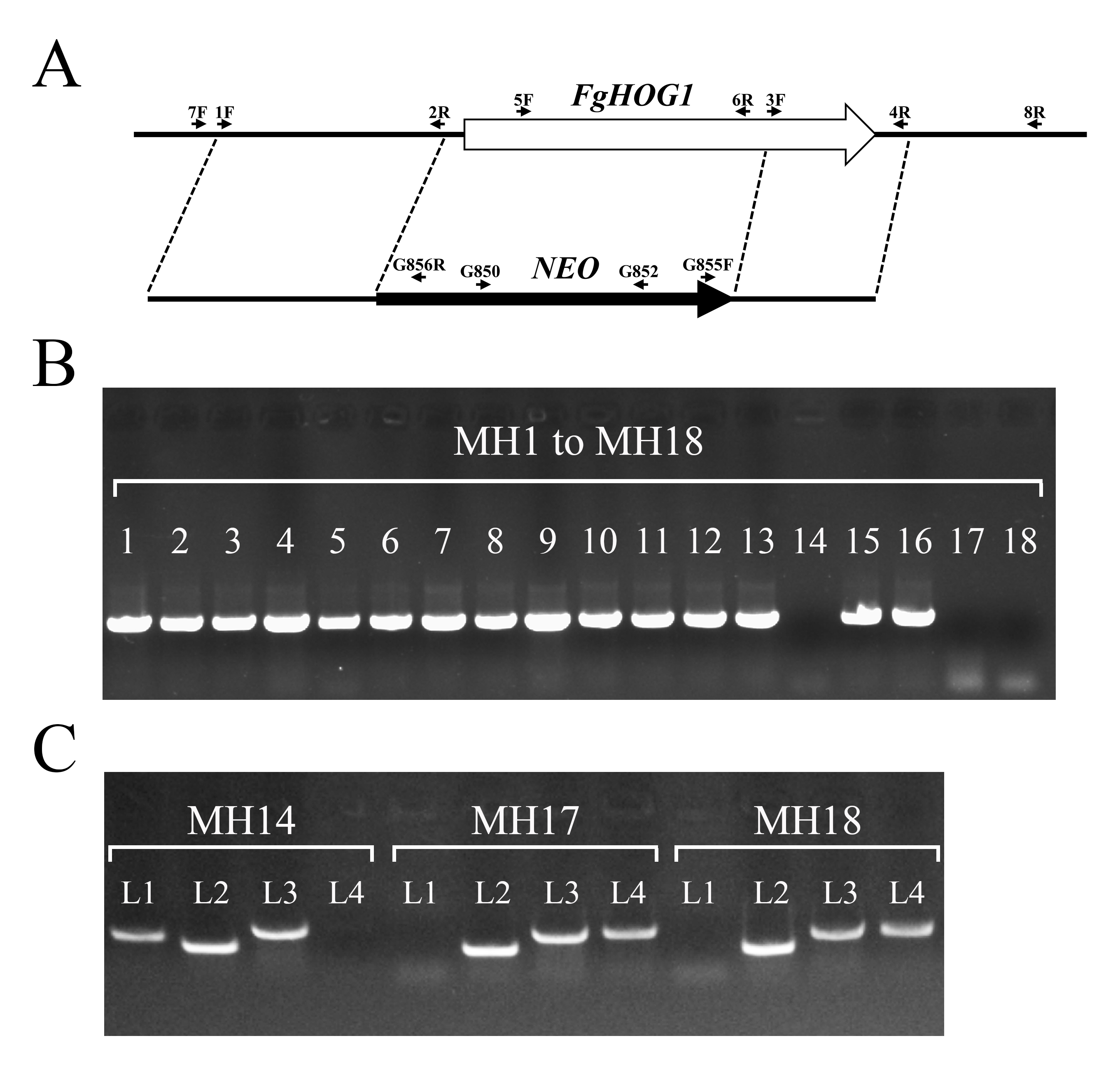

Supplement: FIGURE S1 — PCR assays for the confirmation of the deletion of FgHOG1 under the mgv1 mutant background. (A) The FgHOG1 locus and gene replacement construct. The FgHOG1 and NEO genes are marked with empty and black arrows, respectively. (B) A total of 18 G418-resistant mgv1 Fghog1 transformants (MH1 to MH18) were screened by PCR with primers 5F and 6R. (C) MH14, MH17, and MH18 were further verified by PCR with four pairs of primers, L1 (5F + 6R), L2 (G850 + G852), L3 (7F + G856R), and L4 (G855F + 8R). [file Image_1.JPEG]

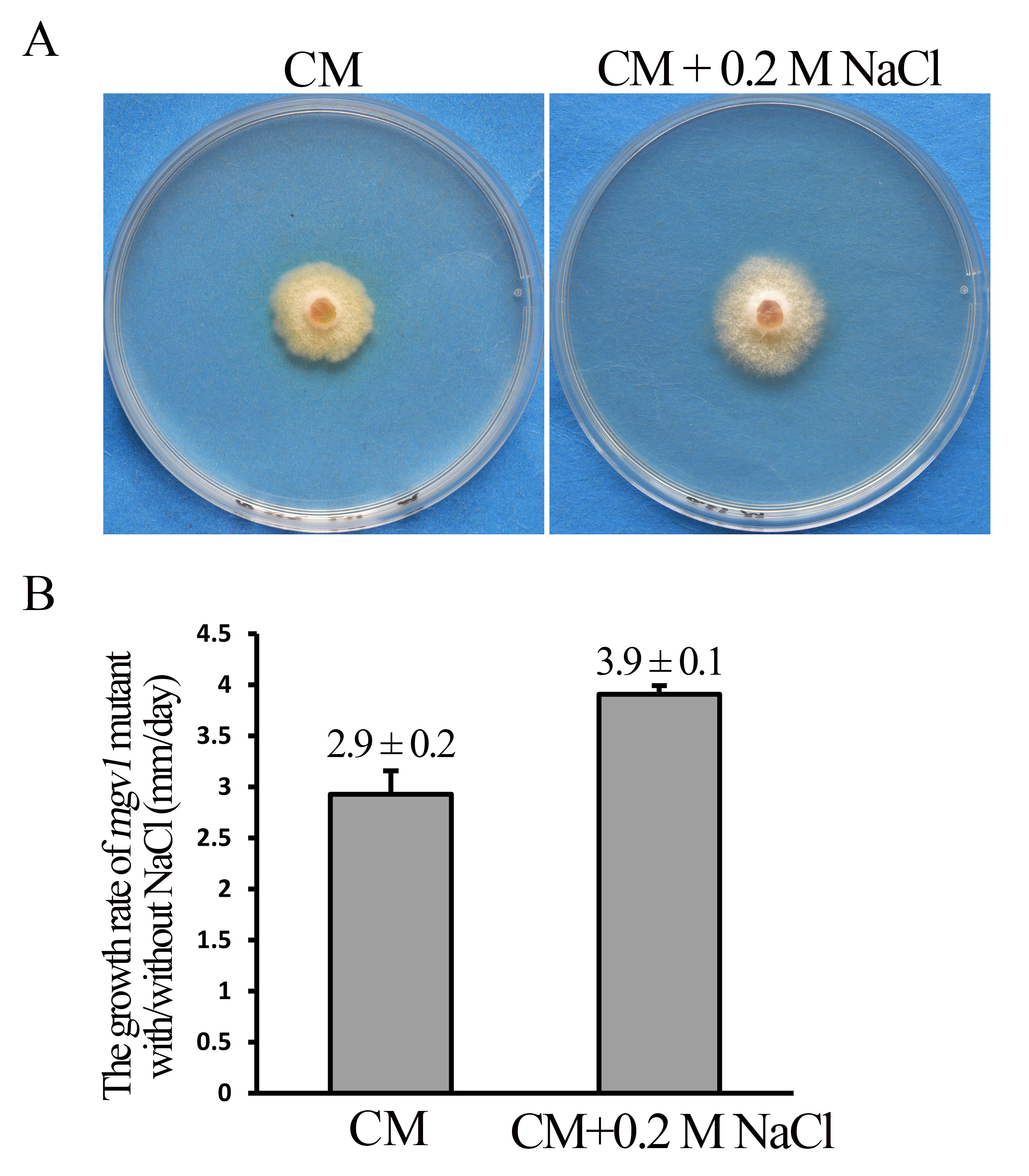

Supplement: FIGURE S2 — The mgv1 mutant grew faster on CM containing 0.2 M NaCl than on regular CM. (A) Three-day-old cultures of mgv1 mutant grown on CM with and without 0.2 M NaCl. The mgv1 mutant grew faster on CM containing 0.2 M NaCl than on regular CM. (B) The diagram shows the growth rate of mgv1 mutant on CM with and 0.2 M NaCl. [file Image_2.JPEG]
